# Esophageal-Crop Capillariasis and Proventriculus Ventriculus Hystrichisiasis in a Migratory Duck (*Anas crecca*)

**Published:** 2019

**Authors:** Mohammad Javad GHARAGOZLOU, Iraj MOBEDI, Reza AGHAEBRAHIMI SAMANI, Fariba TAGHIZADEH, Gholamreza MOWLAVI

**Affiliations:** 1. Department of Pathology, School of Veterinary Medicine, University of Tehran, Tehran, Iran; 2. Department of Medical Parasitology and Mycology, School of Public Health, Tehran University of Medical Sciences,Tehran, Iran; 3. Field Practitioner Veterinarian, Babol, Iran

**Keywords:** *Anas crecca*, *C. contorta*, *H. tricolor*, Infection, Iran

## Abstract

**Background:**

The aim of the present study was to study pathological and parasitological characteristics of simultaneous capillariasis and hystrichiasis in a diseased *Anas crecca* captured in Mazandaran Province of Iran on Oct 2016.

**Methods:**

Parasitology and histopathology techniques were used to detect the parasites of the carcass of the captive common teal.

**Results:**

Macroscopically, severe chronic inflammatory reactions and nodular or granuloma formation and irregular thickening of the affected wall of the esophagus, crop, proventrculus and ventriculus were observed. Microscopically, apart from the chronic inflammatory reactions and granuloma formation, in the paraffin sections stained with Harris Hematoxyline and Eosin technique, the characteristics of the mature female and male *Capillaria* spp. and their barrel-shaped operculated embryonated eggs were found within the tunnels burrowed by the nematode in the hyperplastic stratified squamous epithelium of the inflamed crop and distal portion of the esophagus. The mature female *Hystrichis* spp. containing oval-shaped, embryonated non-operculated eggs and male parasite were found within the labyrinthus spaces formed in the submucosa granulomas of the ventriculus and proventriculus.

**Conclusion:**

Based on the parasitological and pathological studies, the species of the nematode parasites were identified as *Capillaria (Thominx) contorta* and *Hystrichis. tricolor*.

## Introduction

Different species of *Capillaria* nematodes including *C. caudinflata*, *C. obsingata, C. anatis*, *C. annulata* and *C. controta* occur in birds (1). Of the six species, *C. annulata* and *C. contorta* infect esophageal and crop tissues (1–2). As concluded, *C. contorta* is synonymous with *C. annulata* and the correct name for these two species is *C. contorta*. Only a single species of *Hystrichis*, namely *H. tricolor* has been reported to induce proventriculus (1, 2) and esophagus (2) helminth infection in the domestic and wild ducks (1,2). *C. contorta* is a thread-like nematode that invades the stratified squamous epithelium of the esophagus and crop of various avian family and species including domestic poultry, turkey, bobwhite quill, partridge, pheasant and many other domestic and wild birds such as waterfowls.

The parasite was reported in Europe and North America (3). The nematode burrows into the esophagus and crop mucosa. The *Capillaria* eggs within sloughed stratified squamous epithelium appeared in the dropping of the infected bird. The susceptible birds are infected indirectly through ingestion of infected earthworms containing ova or directly through ingestion of the eggs. The nematode induces a detrimental chronic inflammatory reaction in the affected tissues resulting in anorexia and emaciation that can be fatal*. H. tricolor* is a nematode of ducks and wild anatidae birds and it has been reported in Europe and North America (2–4).

The parasite invades the proventriculus, ventriculus and esophagus tissues of birds and it causes inflammation that results in pea or hazelnut nodules or tumor like masses. The susceptible birds that eat the earthworms containing developed *Hystrichis* eggs become infected. The well-developed lesions are host to the mature nematode parasites, intricately attached to the chronic nodular inflammatory lesions induced by the parasites (1).

The aim of the present study was to study pathological and parasitological characteristics of simultaneous capillariasis and hystrichiasis in an *Anas crecca*.

## Materials and Methods

The carcass of a captive common teal (*A. crecca*) from Babol County, Bandpey-ye Sharqi district, located in the Mazandaran Province of Iran was submitted to the Department of Pathology, School of Veterinary Medicine, University of Tehran, Iran on Oct 2016 for disease diagnosis and cause of death. *A. crecca* is a migratory bird which is smaller than its counterpart duck family (*anatidae*), it breeds in Eurasia and migrate towards the south from the Black Sea, Aral Sea, the northern coast of the Caspian Sea during winter and spend the winter period in the lagoons, lakes, ponds and rivers of Iran particularly in areas located in the northern parts of Iran, Gilan and Mazandaran provinces. The body of the bird was covered with a combination of grey and brownish plumages. As described by the veterinarian, prior to death, the bird had manifested clinical signs and symptoms of dysphagia, severe weakness, cachexia and slow unsteady gait as well as inability to fly. The bird did not respond to conventional treatment and died a short period of time after its capture. The carcass of the bird was used for necropsy, the external and internal organs and tissues were inspected for macroscopic changes. Due to the pathological lesions of the proximal parts of the digestive tract, the organ was removed as a whole and placed into 10% buffered formaldehyde solution for histopathological and parasitological studies. Selected tissue samples from the tissues immersed in formalin including the esophagus, crop, proventriculus and ventriculus were processed in an automatic tissue processor. Afterward, paraffin blocks were made and finally, 5–6 μm paraffin tissue sections were stained with the Harris Hematoxyline and Eosin (H&E) technique (5). The H&E stained tissue sections were studied under a light microscope and the diameters of the parasites and their eggs were measured with an eyepiece graticule. For parasitological examination, the nematodes removed carefully from the tissues as much as possible, were stained with 0.2%–1% azocarmine lacto phenol solution for an hour, and then transferred to the Lacto phenol solution (one-part of lactic acid + two parts of glycerin + one part of melted phenol + one part of distilled water ) and finally the parasites were mounted using gelatin-glycerin mounting media (10 gr of gelatin granules was dissolved in 60 ml of distilled water at 75–80 °C, then 70 ml of glycerin was added and heated until the granules dissolved). To prevent fungal contaminations, 0.5 ml of melted phenol crystals per 100 ml of gelatin-glycerin was added (6). The microanatomy of both nematodes was investigated using the paraffin sections stained with the Harris H&E staining technique. The morphological characteristics and the genus and species of the helminth nematodes were identified with the aid of a camera-lucida microscope (Leitz-Wetzlar, S.M. Lux, and Germany), Stereo microscope and Light microscope.

## Results

Based on parasitological examinations, two kinds of nematode helminths, *Capillaria* spp. and *Hystrichis* spp. were diagnosed as causative agents of the proximal parts of the digestive tract lesions and disease.

**Fig. 1:**
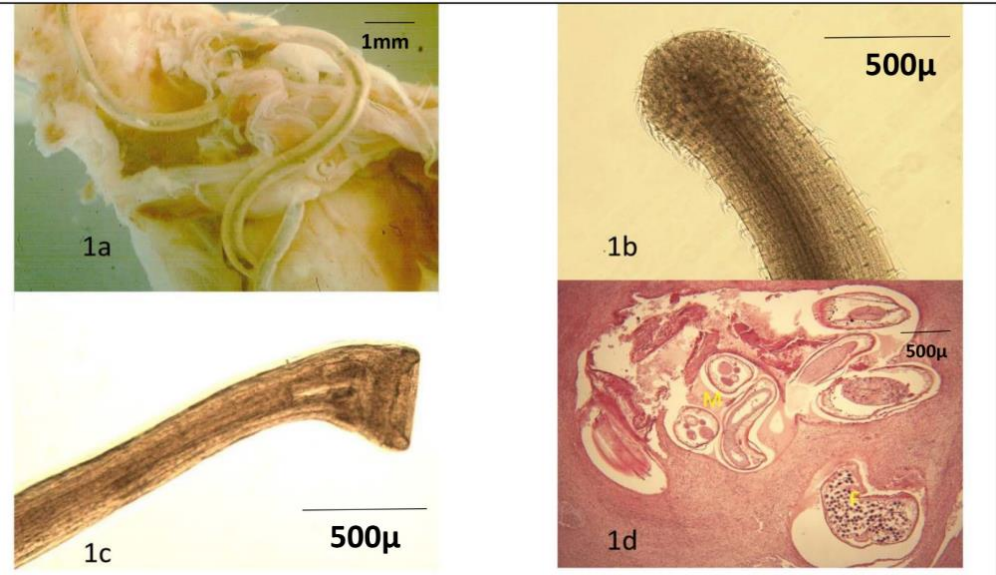
**(a)** A macroscopic view from the inflamed and deformed proventriculus and ventriculus. The *H. tricolor* nematodes are emerging from the serosa surfaces; **(b)** the dilated cephalic ends and its regularly arranged spines in the *H. tricolor*; **(c)** caudal end bell-shaped in the *H. trcolor* and **(d)** H&E stained paraffin section from the ventriculus, the cross-sections of female (F) and male (M) of *H. tricolor* are lodged within cavities or labyrinths formed in the parasite-induced granuloma X100

Prior to the postmortem examination, the bird was cachectic, the body's muscles particularly the pectoral muscle was severely atrophic. The entire gastrointestinal tract was empty and the crop and distal part of the esophagus, the ventriculus (gizzard) and distal part of the proventriculus were severely deformed anatomically. The head and tail of a number of *Hystrichis* spp. nematodes emerged from the inflamed serosa surfaces of the proventriculus and ventriculus (Fig. 1a).

**Fig. 2:**
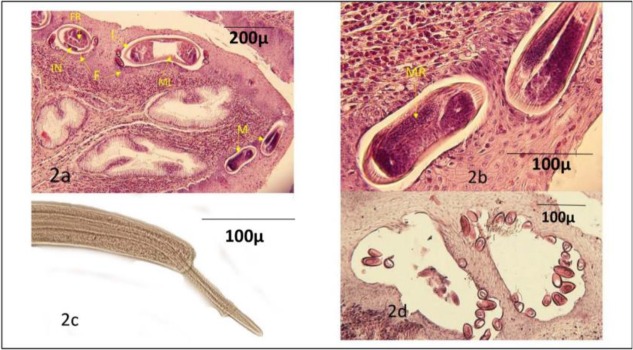
**(a)** The cross-sections of Female (F) and male (M) of *C. contorta* and its barrel-shaped operculated emryonated eggs within tunnels borrowed in the hyperplastic stratified epithelium, integument (I), muscle layer (ML), intestine (IN), male (MR) and female (FR) reproductive organs, H&E, X 100; **(b)** higher magnification of *C. contarta*, the male gamete generating organ containing primordia cells of spermatozoa and mature spermatozoa is evident, H&E, X 400; **(c)** sheathed spicule of *C. contarta*, the sheath is covered with the fine hair-like processes; and **(d)** the higher magnification of *C. cotorta eggs*, the eggshell stained deep purple, H&E X 400

The walls of the esophagus, crop, proventriculus and ventriculus were severely and irregularly thickened. Multiple nodular lesions or granulomas were observed within the walls of the proventriculus and ventriculus. Due to severe inflammatory reactions and other tissue changes, the lumen of the affected part of the digestive tract was narrowed or obliterated. Histopathologically, severe hyperplasia of the distal esophagus epithelium and the crop epithelium, intense chronic inflammatory reactions characterized by infiltration of lymphoid cells, plasma cells, desmoplasia, lymphoid follicles formation and a large number of eosinophils were found in the lamina propria of the affected esophagus and crop infected with *Capillaria* spp. (Fig. 2a).

The cross or oblique sections of the female and male *Capillaria* parasites and released mature barrel-shaped operculated embryonated eggs of size X 50–52.5 X 22.5–25 micrometers were found within the tunnels burrowed in the hyperplastic stratified epithelium of the inflamed esophagus and crop (Fig. 2d). The H&E stained sections of the male *Capillaria* parasite from the outer to inner stained sections were characterized by a non-spinous integument, a delicate and tiny muscle layer, hardly detectable cord cells, digestive tract layered by a basophilic cuboidal epithelium and male gamete producing organ (testes) composed of primordial cells of the spermatozoa and mature spermatozoa (Figs.2a–2b). In addition, the thickness of the wall of the ova was 2.5 μm and it was homogeneously stained dark purple (Fig. 2d). The diameters of the male and female cross-sections at the area of the gonads were 57.5 and 138 μm, respectively. The female parasite was characterized by a non-spinous integument, a delicate tiny muscle layer, hardly detectable lateral cord cells, digestive tract lined by a single layer of basophilic cylindrical cells and female gamete producing organs containing primordial cells of the ova (Fig. 2a).

**Fig. 3:**
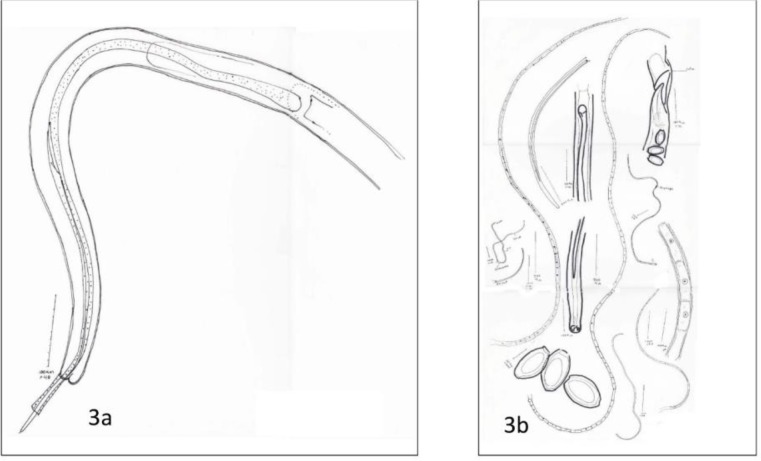
**(a)** The picture of the caudal part of the male *C. contorta* drawn by the aid of Camera Lucida Microscope, the spicule sheath is covered with fine hair-like processes; **(b)** a picture of parasitological characteristics of female and male *C. contorta* and the capillaria eggs drawn by the aid of Camera Lucida Microscope

The male and female characteristics of *C. contorta* drawn by Camera Lucida Microscope are shown in Figs. 3a and 3b respectively.

**Fig. 4:**
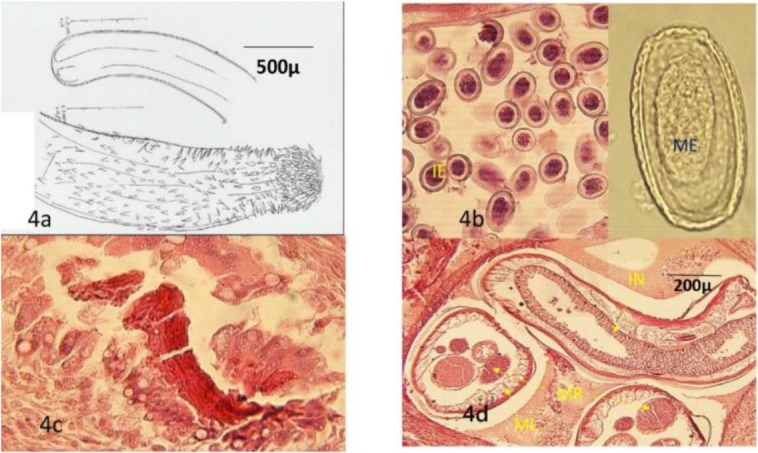
**(a)** A picture from the anterior and posterior ends of *H. tricolor* drawn by the aid of Camera Lucida microscope; **(b)** The formalin-fixed mature eggs (ME), and H&E stained intra uterus developing immature eggs(IE) of *H. tricolor* X400; **(c)** *H. tricolor* induced chronic granulomatous reactions surround the remnants of the death parasite and **(d)** the cross and oblique sections of male(M) and female(F) *H. tricolor*, spiny integument (I), muscle layer (ML), intestine (IN) and male (MR) and female (FR) reproductive organs. H&E, X400

The wall of the proventriculus and ventriculus infected with *Hystrichis* spp. showed labyrinthus granulomatous lesions characterized by a massive desmoplasia, presence of lymphoid cells, plasma cells, some eosinophils and focal areas of accumulation of epithelioid cells, giant cells and a number of lymphoid cells that had surrounded the intensely eosinophilic materials that looks like remains of the dead parasites or ovum (Fig. 4c) as well as presence of the male and female *Hystrichis* spp. within the intercommunicating tunnels so-called labyrinths (Figs. 1d, 4d).

Due to the chronic inflammation, a part of the tunica muscularis of the affected tissues was replaced by the chronic inflammatory tissue. Accumulation of lymphoid cells had replaced a portion of the gizzard and proventriculus mucosa. In addition, there was presence of a few mature *Hystrichis* ova on the affected mucosa surface. Micro anatomically, the male and female parasites consisted of a spinous integument, a thick muscle layer, small lateral cord cells, gut tissue lined by large cuboidal or cylindrical vacuolated epithelium, and the reproductive organ consisted of ovum producing organ and uterus containing developing eggs or immature oval-shaped embryonated and non-operculated eggs of size 27.5–28 × 37.5–40 micrometers (Fig. 4b). The wall thickness of the ova was 3.75 μm and composed of outer thin layer and inner thick layer that was stained dark purple and light brown, respectively. The diameters of the free mature eggs were measured as 90 × 50 micrometers (Fig. 4b). At cross-sections, the diameters of the female and male parasites at the gonadal area were 827 × 460 and 345 × 368 μm, respectively. Parasitologically, a full-sized male *C. contorta* was 12 mm long with long sheathed spicules.

Based on the parasite and its egg's morphology, it was identified as *C. (Thominx) contorta* (Figs. 2c, 2d, 3a, 3b). Only the head and tail of the *Hystrichis* spp. were studied since the mode of the tissue localization and penetration of the nematode prevented complete removal of the parasite. Spindle-shaped head and spinal arrangement on its head (Figs. 1b, 4a) and bell-shaped tail (Figs 1c & 4a) and other characteristics including egg's morphology shown below were used to identify the parasite as *H. tricolor*. The summary of morphological characteristics of the *C. contorta* in the present study are given below:

### Tissue localization and tropism

Stratified squamous epithelium of esophagus and crop is shown in Figs. 2a, 2b, 2d.

### The mature egg size and morphology

Barrel-shaped operculated and embryonated eggs of size 22. 5 – 25 × 50–52.5 micrometer are shown in Figs. 2d, 3b.

### The morphology of the nematode

Thread-like nematode, the full male size was about 12 mm long and 60 μm wide, slender sheathed spicule, the size of the spicule sheath was 800 μm and it was covered with hair like processes (Figs. 2c, 3a). Additionally, there were two terminal laterodorsal prominences on the tail (Figs. 2c, 3a). In spite of the similarities, lack of a cuticular swelling at the back of the head is a hallmark for differentiating C*. contorta* from *C. annulata*.

The summary of morphological characteristics of the *H. tricolor* are given below:

### Tissue localization and tropism

The mucosa, submucosa and serosa of proventriculus and ventriculus (Figs. 1a, 1d, 4d).

### The mature egg size and morphology

Oval-shaped, thick-shelled and non-operculated, embryonated eggs of size 90 × 50 micrometers. The truncated poles of the eggs were covered with the minute tubercles (Fig. 4b).

### The morphology of the nematode

Anterior extremity was swollen and produced several rows of small spines, laid backward (Figs. 1b, 4a), and a bell-shaped posterior end of the male nematode (Fig. 1c).

## Discussion

Based on parasitological and pathological studies and types of tissue tropism, the nematodes were identified as C*. contorta* and *H. tricolor*. In addition to the differences in the morphological characteristics, when compared with *C. contorta* which lives within tunnels burrowed in the hyperplastic mucosa of the esophagus and crop, *H. tricolor* prefer to reside within the intercommunicating cavities or tunnels formed in the parasite-induced granulomatous inflammatory lesions of the submucosa of proventriculus and ventriculus. The tissue tropism of the *C. contorta* and *H. tricolor* is comparable with *Gongylonema pulchrum* and *Draschia (Habronema) megastoma,* respectively (7), and the emergence of the parasite from the serosal surface of the gut is similar to that of wild boar nematode, *Macracanthorhynchus hirudinaceus* (8).

*C. contorta* parasite sequestered itself within the mucosa to evade detrimental immune inflammatory reactions, which in turn enabled the parasite to complete its life cycle. Adversely, *H. tricolor* parasite resides and completes its life cycle within a hostile environment composed of detrimental humoral and cellular factors. The parasite was armed with physiological and biological instruments that neutralized or inhibited the host injurious agents, helping the parasite to survive. The degenerated ova, parasite excretion, released parasite antigens and remains of the dead parasite could induce an intense granulomatous inflammatory reaction that could deteriorate the physiology of the affected organs due to the anatomical and histological deformity, which could gradually increase the severity of the clinical manifestations. *C. contorta* could induce immune inflammatory response in the submucosa of the affected organ when the parasite itself, parasite antigens, and excretions and ova are exposed to the immune inflammatory cellular elements.

Compared to the feeble *C. contorta,* which possess a delicate anatomical and behavioral properties and susceptibility to the host defense mechanisms, due to the strong body conditions including larger body size, strong musculatures, spinous head and the ability to resist the host immune-inflammatory responses, the *H. tricolor* is tremendously an invasive and injurious parasite as can be deduced from the findings of this research paper.

## Conclusion

A chronic inflammatory reaction accompanied by large number of eosinophils occurred. The bird died due to narrowing and obstruction of the inflamed digestive tract. The tissue localization, types of lesions, nematodes' characteristics, eggs' morphology found in the present paper are in complete consonance with the results of other studies related to *Capillaria* spp. and *Hystrichis* spp. infections .To the best knowledge of the authors, the co-infection of these two nematodes, the details of histopathological and parasitological studies of the lesions induced by these nematodes have not been previously reported and studied.
